# Impact of entrepreneurial curriculum on entrepreneurial competencies among students: The mediating role of the campus learning environment in higher education

**DOI:** 10.3389/fpsyg.2022.950440

**Published:** 2022-09-15

**Authors:** Javed Iqbal, Muhammad Zaheer Asghar, Ali Asghar, Yasira Waqar

**Affiliations:** ^1^School of Education, Guangzhou University, Guangzhou, China; ^2^Department of Education, University of Helsinki, Helsinki, Finland; ^3^Department of Education, University of Management and Technology, Lahore, Pakistan; ^4^Doctoral School of ICT and Education (E-Learning), Universitat Oberta de Catalunya, Barcelona, Spain; ^5^Dr Hasan Murad School of Management (HSM), University of Management and Technology, Lahore, Pakistan; ^6^School of Education, Lahore University of Management Sciences, Lahore, Pakistan

**Keywords:** entrepreneurial curriculum, entrepreneurial competencies, campus learning environment, measurement model, structural equation model, higher education

## Abstract

This study explored the direct and indirect influences of the entrepreneurial curriculum on entrepreneurial competencies, using the campus learning environment as a mediator. In this study, a survey questionnaire composed of 48 items was used to collect data on the entrepreneurial curriculum, entrepreneurial competencies, and campus learning environment from pre-service vocational teachers enrolled in six universities located in Hunan Province, China. The entrepreneurial curriculum has four components, namely, curriculum content, curriculum material, teaching strategies, and feedback and assessment. Partial least squares structural equation modeling was used through SmartPLS 3.3.3 to measure the effects. The curriculum content has a direct, significant, and positive influence on entrepreneurial competencies. For the indirect influence, all four dimensions of the entrepreneurial curriculum influenced the campus learning environment, which, in turn, was positively associated with entrepreneurial competencies. The campus learning environment was therefore revealed to play a mediating role between the entrepreneurial curriculum and entrepreneurial competencies. The study explored that effective entrepreneurial curriculum delivery and campus learning environment are helpful for developing entrepreneurial competencies among the pre-service vocational teachers. Universities should take initiatives to update the entrepreneurial curriculum and create a conducive campus learning environment that brings a positive change to develop entrepreneurial competencies among their students. Moreover, practical implications and future research directions are also discussed in this article.

## Introduction

Entrepreneurship education is an emerging trend to develop an entrepreneurial mindset worldwide (Fayolle, 2018; Huang et al., 2020). Entrepreneurship is considered the backbone of a country’s economy; therefore, more than 3,000 universities worldwide offer multiple degrees, courses, and certifications to produce graduates with entrepreneurial competencies and mindsets (Turner and Gianiodis, 2018). Entrepreneurship competency development depends on an integrated entrepreneurial curriculum among graduates (Kwong and Thompson, 2016). Several higher education institutions have incorporated the entrepreneurial curriculum into different degree programs with a significant focus on science, engineering, technology, humanities and arts, medical sciences, and life sciences (Turner and Gianiodis, 2018). The literature on the entrepreneurial competencies of students within the vocational teaching discipline remains exceptionally limited.

The entrepreneurial curriculum emphasizes updating the curriculum content and material, teaching strategies, and feedback and assessment practices to prepare graduates to become self-employees after graduation (Premand et al., 2016; Iwu et al., 2019). The quality entrepreneurial curriculum content helps students establish new organizations and become promising entrepreneurs in future (Katundu and Gabagambi, 2016; Khorrami et al., 2018). However, despite various educational entrepreneurship programs, graduates still scarcely show a career in entrepreneurship or hardly consider becoming entrepreneurs (Rudhumbu et al., 2016). The solution to this problem lies under the improvement of curriculum components, such as curriculum content and material, teaching strategies, and feedback and assessment, because these components are considered the predictors of entrepreneurial competencies (Iwu et al., 2019). Therefore, this study explored how the entrepreneurial curriculum influences entrepreneurial competencies among students.

The literature describes various types of entrepreneurial competencies that may be accelerated through the entrepreneurial curriculum. This study aimed to analyze three possibly teachable entrepreneurial competencies, namely, networking competency, proactiveness, and conceptual understanding collectively (Morris et al., 2017). Each of these competencies is significant for fostering an entrepreneurial attitude among students. Specifically, theories of entrepreneurship education provide the foundations to establish these competencies (Morris et al., 2015). It was assumed that entrepreneurial competencies are improved through implementation of the entrepreneurial curriculum and campus learning environment (Matlay et al., 2015; Belitski and Heron, 2017; Bischoff et al., 2018). Therefore, this study also realistically explored the campus learning environment effect on entrepreneurial competencies.

There has been criticism of the sort of students produced by higher education institutions, who may lack the necessary abilities for the demands of the modern organizational environment (Kwong et al., 2012). Knowledge-based economy increases the demand for graduates with a specific area of competencies across a wide range of entrepreneurial education. It is assumed that there is supplemented demand of the graduates who can work in a dynamic environment with their creative mindset. An entrepreneurial campus learning environment has been identified as being able to fill this gap through offering various exposures inside and outside of the classroom, such as creating intellectual interest, in-depth knowledge, teacher–student interaction, pedagogy practice, and utilizing resources to build information communication technology capabilities among future entrepreneurs (Fayolle and Gailly, 2015; Lee et al., 2018). Thus, the literature provides the foundation to explore the influence of the entrepreneurial curriculum and campus learning environment on entrepreneurial competencies (Yin and Wang, 2017; Maxwell et al., 2018; Heaton et al., 2019). The existing literature suggested to explore the connection between the entrepreneurial curriculum and entrepreneurial competencies through the campus learning environment. Therefore, we assumed that the campus learning environment has a mediating role between the entrepreneurial curriculum and entrepreneurial competencies. Thus, the present study explores the mediating role of the campus learning environment between entrepreneurial competencies and the entrepreneurial curriculum.

Despite the high level of interest among scholars and practitioners, there are still significant gaps in understanding the connections between the entrepreneurial curriculum, campus learning environment, and entrepreneurial competencies. First, this type of research is mostly conducted in advanced countries, such as Finland, the United States, Australia, South Korea, and the United Kingdom (Seikkula-Leino, 2011; QAA, 2012; Lee and Park, 2014; Choi and Markham, 2019; Maritz et al., 2019). Entrepreneurship education research results are meaningful in emerging nations to produce quality entrepreneurs. Second, in the higher education sector, academic research focuses on changing and updating the entrepreneurial curriculum and campus learning environment to develop entrepreneurial competencies among their students. Third, fewer research studies focus on exploring the connections between the entrepreneurial curriculum, campus learning environment, and entrepreneurial competencies; however, a three-way connection between the entrepreneurial curriculum, campus learning environment, and entrepreneurial competencies was also yet to be explored. Particularly, researchers have not measured the campus learning environment as a mediating construct between the entrepreneurial curriculum and entrepreneurial competencies.

Based on the aforementioned research motivation, this study aims to address these gaps through a synthesized research framework. This study emphasizes the intervening construct of the campus learning environment, and multiple dimensions of the entrepreneurial curriculum (content, material, teaching strategies, and feedback and assessment) were used in this study. Within this synthesized research framework, the study addressed the following two research questions (RQs):

RQ1: Do entrepreneurial curriculum and campus learning environment influence entrepreneurial competencies?

RQ2: Does campus learning environment mediates the relationship between entrepreneurial curriculum and entrepreneurial competencies?

Section “Literature review” deals with the background, conceptual framework, and hypothesis. Section “Theoretical framework and hypotheses formulation” comprises methodology. Section “Methodology” explains the analysis and interpretations. Section “Data analysis” discusses the results and presents the conclusions. Section “Descriptive statistics” presents implications and future research directions.

## Literature review

### Entrepreneurial curriculum in China

In China, universities are becoming aware of the significance of entrepreneurship education. Recent studies have examined the practical and theoretical issues of designing the entrepreneurial curriculum in Chinese universities (Saklofske et al., 2003). Furthermore, Dou et al. (2019) explained that entrepreneurship education in Chinese universities and the entrepreneurial curriculum have a positive role in enhancing the entrepreneurial attitude and competencies among students. It was also identified a three-factor model from Chinese entrepreneurship education, which included the curriculum, environment, and social environment resources. Universities in China were implementing necessary subjects for entrepreneurship education. Universities also implemented an integrated curriculum in their various degree programs, which are highly positively correlated with the development of an entrepreneurial mindset of the graduates, but it may have still failed to lower the employment pressure (Lin and Xu, 2017). The whole situation provoked the researchers and academicians to explore the real problems regarding entrepreneurship education, including entrepreneurial curriculum, campus learning environment, and entrepreneurial competencies in the Chinese context of higher education.

### Entrepreneurial curriculum

The entrepreneurial curriculum is defined as the subject-specific nature of curriculum material, content, teaching strategies, and feedback and assessment practices utilized by universities to promote students’ entrepreneurial skills, behaviors, attitudes, and competencies (GEM, 2005). The curriculum content is the range of topics (both from theory and practice) that are aligned to develop entrepreneurial competencies among students (Kazakeviciute et al., 2016). The curriculum material includes resources required to deliver a specific curriculum. It includes textbooks, lecture notes, and other AV aids. Teaching strategies are methods used to transmit the curriculum content (Torres-Barreto et al., 2020). The feedback and assessment practices are an important component of curriculum instructions for the development of entrepreneurship competencies. They involve assessing the whole program to judge its effectiveness in developing entrepreneurial competencies among students (Kazakeviciute et al., 2016). Several studies indicated that the entrepreneurial curriculum needs unique content, material, teaching strategies, feedback, and assessment approaches to engage students to improve their entrepreneurial competencies (Plumly et al., 2008). Aghajani and Abbasgholipour (2012) explored that an entrepreneurial curriculum must challenge the students to increase their responsibilities and hardworking spirit to start their own venture after their graduations. Kirby et al. (2011) emphasized that students learn how to apply learned concepts in real entrepreneurial conditions taught through the entrepreneurial curriculum, which ultimately builds entrepreneurial competencies.

### Entrepreneurial competencies

Based on an extensive literature review on entrepreneurial competencies, we defined entrepreneurial competencies as the set of behavioral predispositions that influence students’ abilities to succeed in an entrepreneurial venture (Lee et al., 2018). In this study, entrepreneurial competencies are defined in three dimensions: networking skills, conceptual understanding competencies, and proactivity (Morris et al., 2017). Networking skills is the most important competency in entrepreneurial ventures, where entrepreneurs maintain and build new relations with others stakeholders. In Raza et al. (2018), conceptual understanding competencies are defined as collecting learned patterns, repeated behaviors, and high-order competencies that deal with the turbulent business environment. Proactivity is defined as opportunity-seeking behavior patterns and the tendency to push an idea (Morris et al., 2017; Raza et al., 2018). Therefore, as an important point of explanation, this study is designed with the presumption that the entrepreneurial curriculum and campus learning environment are likely to impact students’ entrepreneurial competencies.

### Campus learning environment

In this study, we defined the campus learning environment as it offers various exposure inside and outside of the classroom, such as creating intellectual interest, provision of in-depth knowledge, teacher–student interaction, pedagogy, resources, and information communication technology resources to develop entrepreneurial competencies (Fayolle and Gailly, 2015; Lee et al., 2018). The campus learning environment offers maximum learning opportunities related to developing entrepreneurial competencies through various objects, such as activities, actions, policies, ecological environment, and psychology (Bogatyreva et al., 2019; Grinevich et al., 2019; Yi, 2020). It was assumed that further research was required to build more in-depth understanding on the role of the campus learning environment to enhance entrepreneurial competencies among students. Therefore, this study explored the mediating role of the campus learning environment between the entrepreneurial curriculum and entrepreneurial competencies.

## Theoretical framework and hypothesis formulation

### Research framework

The literature review revealed that entrepreneurial curriculum dimensions (curriculum content and material, teaching strategies, and feedback and assessment) enhance entrepreneurial competencies through the campus learning environment. The entrepreneurial curriculum and learning environment are the main areas of interest for researchers and teachers due to their role in developing entrepreneurial competencies. The entrepreneurial curriculum is being treated as a core foundation of entrepreneurial competencies (Maxwell et al., 2018) with a mediating role of the campus learning environment (Miller and Acs, 2017). The theory of planned behavior and the human capital theory assert that implementing an entrepreneurial curriculum transforms entrepreneurial competencies (Ajzen, 1991). Similarly, an entrepreneurial curriculum influences student learning in a dynamic higher education environment and constitutes essential tools for their entrepreneurial competencies. Therefore, the current study emphasizes that a campus learning environment is likely to facilitate the relationship between the entrepreneurial curriculum and entrepreneurial competencies. In addition, this study examined the impact of the entrepreneurial curriculum on entrepreneurial competencies through the campus learning environment in an emerging country.

**FIGURE 1:**
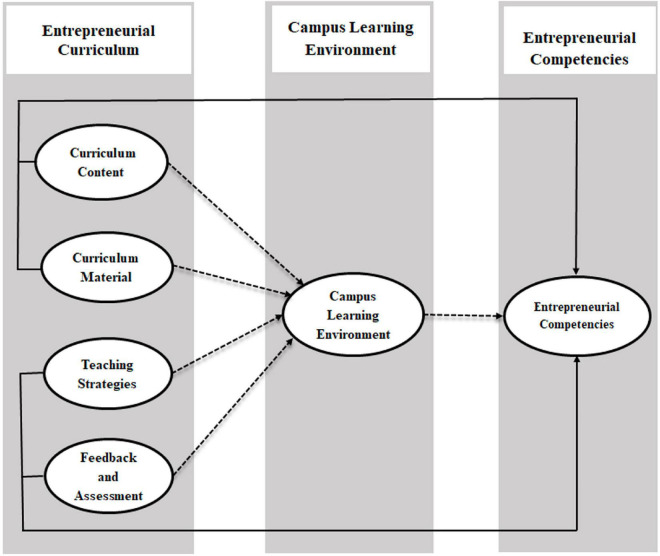
Conceptual framework.

Furthermore, the present study supports the previous literature by clarifying the impact of the entrepreneurial curriculum and dimensions on shaping the campus learning environment and leading to entrepreneurial competencies. It has been established that students can learn entrepreneurial competencies by using campus resources effectively when they are in the university. Entrepreneurial competencies have been described as individuals’ abilities to start their work (De Massis et al., 2018). However, entrepreneurial competencies might also be evaluated related to entrepreneurial intentions. Therefore, universities have common practice to carefully examine their entrepreneurial curriculum in achieving entrepreneurial competencies. Universities are required to ensure a quality integrated curriculum across the programs offered to undergraduates and graduates. It is challenging for universities to develop entrepreneurial competencies among students through an entrepreneurial curriculum, except for the inclusive strategy that may enable universities to confront this challenge (Tanveer and Haq, 2021). Previous literature clarifies that the entrepreneurial curriculum has an important relationship with the campus learning environment and entrepreneurial competencies among students (Collins et al., 2004; Lee et al., 2018). The main purpose of an entrepreneurial curriculum is to develop entrepreneurial competencies through a campus learning environment in higher education. However, universities are also in dire need of an entrepreneurial curriculum that should be an effective and competent faculty to teach and execute this curriculum (Tanveer and Haq, 2021). Based on the literature and theory of planned behavior and human capital theory, scholars like Heuer and Kolvereid claim that students’ entrepreneurial competencies with an effective entrepreneurial curriculum have deep-rooted influences on motivation to start their work activity after graduation (2014). Furthermore, a university entrepreneurial curriculum through a campus learning environment helps the students to improve their entrepreneurial competencies. Based on these discussions, the proposed theoretical model of the study is presented in Figure 1 that presents all hypotheses.

### Entrepreneurial curriculum and entrepreneurial competencies

Multiple studies on entrepreneurship have highlighted the positive connection between the entrepreneurial curriculum (curriculum content, material, teaching strategies, and feedback and assessment) and entrepreneurial competencies (Seikkula-Leino, 2011; Brush, 2014; Esmi et al., 2015; Kummitha and Majumdar, 2015; Marques et al., 2018; Sánchez-Barrioluengo and Benneworth, 2019). Researchers have designed and published studies focused on the entrepreneurial curriculum and its effects on entrepreneurial competencies (Chen et al., 2015). Researchers with the common approach claim that an effective entrepreneurial curriculum improves entrepreneurial competencies (Heuer and Kolvereid, 2014). Others suggest that the entrepreneurial curriculum content influences entrepreneurial, critical thinking abilities (Olokundun et al., 2017). Furthermore, the theory of planned behavior and human capital theory explain entrepreneurial attitude, which is a positive factor that influences entrepreneurial competencies (Heuer and Kolvereid, 2014). The need for changes in the critical entrepreneurial curriculum for developing entrepreneurial mindset has been recognized (Akhmetshin et al., 2019). Similarly, Tanveer and Haq (2021) suggested revisions in the entrepreneurial curriculum to improve entrepreneurial competencies among students. These findings have significantly assisted in the dire need of more understanding of the relationships between the entrepreneurial curriculum and entrepreneurial competencies. Therefore, a positive relationship between the entrepreneurial curriculum (curriculum content and material, teaching strategies, and feedback and assessment) and entrepreneurial competencies is assumed in the following hypotheses:

**H1a:** Entrepreneurial curriculum (curriculum content) positively influences entrepreneurial competencies.

**H1b:** Entrepreneurial curriculum (curriculum material) positively influences entrepreneurial competencies.

**H1c:** Entrepreneurial curriculum (teaching strategies) positively influences entrepreneurial competencies.

**H1d:** Entrepreneurial curriculum (feedback and assess ment) positively influences entrepreneurial competencies.

### Entrepreneurial curriculum and campus learning environment

Researchers have developed theoretical models and related concepts to connect the entrepreneurial curriculum (curriculum content and material, teaching strategies, and feedback and assessment) with the campus learning environment. These studies support the notion that the entrepreneurial curriculum positively correlates with the campus learning environment (Arranz et al., 2017; Igbokwe-Ibeto et al., 2018; Akhmetshin et al., 2019; Gieure et al., 2019; Tanveer and Haq, 2021). The literature consistently argues that university curriculum affects students’ academic performance (Kazakeviciute et al., 2016). Also, an integrated curriculum has created students’ interest to be more efficient, whereas they get exposure from the campus learning environment, which can increase academic performance (Tanveer and Haq, 2021)_ENREF_31. The entrepreneurial curriculum is in the university environment, which prepares and inspires students to start their work activity after graduation (Aghajani and Abbasgholipour, 2012). These arguments suggest that an entrepreneurial curriculum is associated with the campus learning environment. Therefore, the present study explores the relationship between the entrepreneurial curriculum and campus learning environment. We assumed these relationships in the following hypotheses:

**H2a:** Entrepreneurial curriculum (curriculum content) influences the campus learning environment.

**H2b:** Entrepreneurial curriculum (curriculum material) influences the campus learning environment.

**H2c:** Entrepreneurial curriculum (teaching strategies) influences the campus learning environment.

**H2d:** Entrepreneurial curriculum (feedback and assessment) positively influences the campus learning environment.

### Campus learning environment and entrepreneurial competencies

A number of studies highlighted the positive association between campus learning environment and entrepreneurial competencies (Pihie and Bagheri, 2013; Gieure et al., 2019; O’Reilly et al., 2019). The campus learning environment is the antecedent of entrepreneurial competencies (Ibrahim and Lucky, 2014). The theory of planned behavior and human capital theory endorse the approaches that universities education may create opportunities to enhance entrepreneurial competencies (Gür et al., 2017; Hahn et al., 2017). The campus learning environment pushes the students toward learning entrepreneurial competencies (Rasmussen and Borch, 2010). This study indicates that the campus learning environment has a significant and positive association with entrepreneurial competencies. The study predicts that the campus learning environment positively influences entrepreneurial competencies, which results in the following hypothesis:

**H3:** Campus learning environment positively influences entrepreneurial competencies.

### Mediation effect of campus learning environment

Studies reviewed earlier describe that the campus learning environment in various forms directly affects entrepreneurial competencies (Davey, 2015). Azahari Ramli et al. (2018) suggest that the campus learning environment, in terms of resources, act as a mediating factor between entrepreneurial education and entrepreneurial competencies. Similarly, Ibrahim and Lucky (2014) found that the campus learning environment has a positive role in enhancing entrepreneurial competencies.

In addition, researchers have also explored the mediating role of the campus learning environment in the relationship between the entrepreneurial curriculum and entrepreneurial competencies. Luo et al. (2022) conducted a study with 1,100 students in Guangdong, China, and found that the entrepreneurial environment is “the basis for the entrepreneurial project execution and can provide sufficient external conditions for the entrepreneurial activity implementation and concluded that a positive entrepreneurial environment can provide rich resources to support entrepreneurial activities and meet the elemental conditions required for the development of entrepreneurial projects.” Furthermore, Valencia-Arias et al. (2022) found that university environments equipped with tools that facilitate training and the development of entrepreneurial skills using different types of resources allow students to have better attitudes toward entrepreneurship. A campus environment that promotes entrepreneurship through experiential learning is shown to have positive outcomes on students’ career aspirations and entrepreneurial abilities, through various studies, including those by Barnes and de Villiers Scheepers (2018) and Luo et al. (2022). Similarly, Iqbal et al. (2022b) conducted a study on students of higher education and found that the curriculum along with the knowledge of ICT enhanced the entrepreneurial competencies among the students. Another study found that the curriculum like curriculum content, material, and teaching strategies enhanced student outcomes, such as ICT competencies (Ashraf et al., 2022). The authors of the present study thus affirm from findings of the aforementioned literature that the campus learning environment works as a mediator between the entrepreneurial curriculum and entrepreneurial competencies. Therefore, this study explores the mediating role of the campus learning environment between the entrepreneurial curriculum and entrepreneurial competencies. Hence, the following hypotheses are put forward:

**H4a:** Campus learning environment mediates the relationship between entrepreneurial curriculum (curriculum content) and entrepreneurship competencies.

**H4b:** Campus learning environment mediates the relationship between entrepreneurial curriculum (curriculum material) and entrepreneurship competencies.

**H4c:** Campus learning environment mediates the relationship between entrepreneurial curriculum (teaching strategies) and entrepreneurship competencies.

**H4d:** Campus learning environment mediates the relationship between entrepreneurial curriculum (feedback and assessment) and entrepreneurship competencies.

## Methodology

### Design of the research

The cross-sectional survey research design was used for the execution of this study (Iqbal et al., 2022a). We collected the data on entrepreneurial curriculum, campus learning environment, and entrepreneurial competencies by applying a survey questionnaire technique in the target population of pre-service vocational teachers enrolled in the six universities in Hunan, China. All respondents participated voluntarily. We applied the measurement modeling technique to ensure the validity and reliability of the questionnaire. Descriptive statistical analyses were used to describe the demographic characteristic of the respondents. Moreover, PLS-SEM 3.3.3 (the partial least squares structural equation modeling) was used through SmartPLS statistical software to analyze the effects of the entrepreneurial curriculum on entrepreneurial competencies and the campus learning environment as mediators (Chen et al., 2022; Jebbouri et al., 2022). It was assumed that the study would expose a significant relationship between the variables: entrepreneurial curriculum, campus learning environment, and entrepreneurial competencies.

### Research participants

This quantitative research explored the impact of entrepreneurial the curriculum on entrepreneurial competencies with the mediating role of the campus learning environment in China. A stratified random sampling technique was used for selection of participants. The stratified random sampling technique involves dividing the population into smaller sub-groups based on respondents’ shared attributes or characteristics. The target population of this research consisted of pre-service vocational teachers (undergraduates, graduates, and post-graduates). Before we began the final data collection, a pilot study was conducted to validate the instruments, and the necessary data and trends were obtained. We collected the data from vocational teacher education departments in the six universities of Hunan Province. We circulated 500 questionnaires among the targeted population from January 2021 to February 2021 and received 470 questionnaires, while nine questionnaires were found incomplete. Hence, the total useable sample size was 461 pre-service vocational teachers, resulting in a response rate of 92.2%.

### Instrumentation

In this research, entrepreneurial curriculum components (curriculum content, curriculum material, teaching strategies, and feedback and assessment) were independent variables, and entrepreneurial competencies is the dependent variable, while the campus learning environment remained the mediator. The first part of the instrument contained the description of research purposes and guidelines for responding to secrecy and privacy statements. The second part of the instrument consists of participants’ demographic information, such as gender, education level, age, and CGPA. The third part of the instrument describes the 36 items used for selected variables’ items on a seven-point Likert scale (1, strongly disagreed to 7, strongly agreed). The pilot study was conducted on 30 students with same demographic characteristics as the final data sample must check analysis bias. The pilot study was conducted on 30 students with same demographic characteristics as the final data sample had, however, these respondents were not included in final sample. The modified questionnaire was administered for final data collection (see Appendix 1).

### Variables measures

#### Curriculum contents

The five items of the questionnaire related to the curriculum content were adapted and modified from the work of TLEMU-HKU (2018). The responses were recorded on a seven-point Likert scale (1, strongly disagree to 7, strongly agree). The sample items of the questionnaire included “The teachers made it clear right from the start that they expect us to become entrepreneurs” and “the teacher provides detailed information about entrepreneurship competencies.” Cronbach’s alpha value for the entrepreneurial curriculum (curriculum content) was 0.763 (see Table 1), and the standard Cronbach’s alpha index was 0.070 or higher, which meets the threshold criteria.

**TABLE 1:** Reliability and convergent validity.

Constructs	IL	CA	rho_A	CR	AVE
Curriculum content (CC)		0.763	0.763	0.84	0.513
CC1	0.708				
CC2	0.696				
CC3	0.715				
CC4	0.724				
CC5	0.737				
Curriculum material (CM)		0.882	0.883	0.908	0.585
CM1	0.727				
CM2	0.780				
CM3	0.742				
CM4	0.810				
CM5	0.796				
CM6	0.758				
CM7	0.739				
Teaching strategies (TS)		0.892	0.892	0.917	0.649
TS1	0.808				
TS2	0.892				
TS3	0.813				
TS4	0.826				
TS5	0.819				
TS6	0.794				
Feedback and assessment (FA)		0.889	0.891	0.915	0.643
FA1	0.808				
FA2	0.850				
FA3	0.798				
FA4	0.760				
FA5	0.769				
FA6	0.824				
Campus learning environment (CLE)		0.805	0.808	0.86	0.506
CLE1	0.722				
CLE2	0.719				
CLE3	0.738				
CLE4	0.677				
CLE5	0.727				
CLE6	0.664				
CLE7	0.738				
Entrepreneurial competencies (EC)		0.939	0.940	0.946	0.506
EC1	0.656				
EC2	0.690				
EC3	0.663				
EC4	0.692				
EC5	0.680				
EC6	0.713				
EC7	0.750				
EC8	0.745				
EC9	0.739				
EC10	0.755				
EC11	0.742				
EC12	0.799				
EC13	0.722				
EC14	0.718				
EC15	0.702				
EC16	0.670				
EC17	0.738				

IL, indicator loading, alpha, Cronbach’s alpha; CR, composite reliability; AVE, average variance extracted.

#### Curriculum material

The seven items related to curriculum material were adapted and modified from the work of TLEMU-HKU (2018). The responses were recorded on a seven-point Likert scale (1, strongly disagree to 7, strongly agree). The sample items of the questionnaire included “our teachers use material of entrepreneurial education that enhances my motivation to develop entrepreneurial competencies” and “our teacher uses audio, video, and well-devised material about entrepreneurship in the class.” Cronbach’s alpha index was 0.882, and the standard Cronbach’s alpha index was 0.070 or higher that met the threshold criteria (see Table 1).

#### Teaching strategies

The six items related to teaching strategies were adapted and modified from the work of TLEMU-HKU (2018). The responses were recorded on a seven-point Likert scale (1, strongly disagree to 7, strongly agree). The sample items of the questionnaire included “our teacher keeps me motivated and engaged in entrepreneurial learning” and “our teacher uses various teaching strategies during teaching.” Cronbach’s alpha value for the entrepreneurial curriculum (teaching strategies) (0.892) and the standard Cronbach’s alpha index (0.070 or higher) have met the threshold criteria (see Table 1).

#### Feedback and assessment

The six items related to feedback and assessment were adapted and modified from the work of TLEMU-HKU (2018). The responses were recorded on a seven-point Likert scale (1, strongly disagree to 7, strongly agree). The sample items of the questionnaire included “our teachers give constructive feedback on my progress” and “the teachers invest much time into commenting on my work.” Cronbach’s alpha (0.889) and the standard Cronbach’s alpha index (0.070 or higher) have met the threshold criteria (see Table 1).

#### Campus learning environment

The seven items related to the campus learning environment were adapted and modified from the work of TLEMU-HKU (2018). The responses were recorded on a seven-point Likert scale (1, strongly disagree to 7, strongly agree). The sample items of the questionnaire included “I am able to discuss topics of broader intellectual interest with teachers about entrepreneurship,” and “my teachers provide opportunities for interaction in class.” Cronbach’s alpha value for the campus learning environment (0.805) and the standard Cronbach’s alpha index (0.070 or higher) have met the threshold criteria.

#### Entrepreneurial competencies

The 17 items related to entrepreneurial competencies were adapted and modified from the work of Man (2001). The responses were recorded on a 7-point Likert scale (1, strongly disagree to 7, strongly agree). The sample items of the questionnaire included “after completing my education program, and I would make a rational decision in the organization and negotiate with others.” Cronbach’s alpha (0.939) and standard Cronbach’s alpha index (0.70) have met the threshold criteria.

#### Demographics

Selected universities develop entrepreneurial competencies among students (prospective educational entrepreneurs) by creating a supportive learning environment and effective curriculum delivery through the effective and integrated entrepreneurial curriculum. The samples’ demographic characteristics included gender, background, age, education level, and CGPA. There were 461 respondents, representing male 121 (26.2%) and female 340 (73.8%); rural 197 (42.7) and urban (57.3%); less than 22 years, 279 (60.5%), 23–30 years, 165 (35.8%), and above 30 years, 17 (3.7%); undergraduate 159 (34.5), graduate 252 (54.7%), and post-graduate 50 (10.8%); and respondents having less than 2.00 CGPA 3 (.7%), 2.00–3.00 125 CGPA (27.7%), 3.10–3.50 CGPA 233 (50.5%), and 3.51–4.00 CGPA 100 (21.7%). The details of the demographics of sample are exhibited in Table 2.

**TABLE 2:** Participants’ demographics.

Measure	Items	Frequency (n)	Percentage (%)
Gender	Male	121	26.2
	Female	340	73.8
	Total	461	100.0
Background	Rural	197	42.7
	Urban	264	57.3
	Total	461	100.0
Age	Less than 22	279	60.5
	23–30	165	35.8
	Above 30	017	3.70
Education level	Undergraduate	159	34.5
	Graduate	252	54.7
	Post-graduate	050	10.8
	Total	461	100.0
CGPA	Less than 2.00	003	0.70
	2.00–3.00	125	27.7
	3.10–3.50	233	50.5
	3.51–4	100	21.7
	Total	461	100.0

## Data analysis

The PLS-SEM evaluates partial model structures by merging principal component analysis with ordinary least squares regressions (Mateos-Aparicio, 2011). The study used the partial least squares structural equation modeling (PLS-SEM 3.2.2) method to investigate direct and indirect effects used in the theoretical framework (Rasool et al., 2019). For this study, we selected PLS-SEM to analyze our study’s complex structural modeling. Our research is composed of reflective scales. PLS-SEM deals with both small and large sample sizes. Moreover, it works well with distribution issues, such as lack of normality (Hair et al., 2019; Rasool et al., 2019).

We began data analysis by applying measurement modeling to ensure the reliability and validity of scales (curriculum objective, teaching strategies, feedback and assessment, campus learning environment, and entrepreneurial competencies). Reflective measurement models were tested on indicator loading, Cronbach’s alpha, rho_A, composite reliability, and average variance extracted (AVE) for convergent validity. The indicator loading threshold value is more than 0.60. The threshold value for Cronbach’s alpha, rho_A, and composite reliability is 0.70 on each construct. The AVE-required threshold value is 0.50. Table 1 indicates that reliability indicators showed the required level of the indexes, such as indicator loading > 0.60, Cronbach’s alpha > 0.70, rho_A > 0.70, and composite reliability > 0.70, which means the instrument was reliable. Table 1 also indicates that the AVE above 0.50 means the instrument fulfilled the convergent validity requirements. It was concluded that the instrument was reliable and valid. The detailed results are also presented in Table 1.

Convergent validity was measured by calculating the average variance extracted (AVE) at the threshold value of 0.50 for all items on each construct. Table 3 shows that AVE values for each construct are higher than the threshold value. Furthermore, the heterotrait-to-monotrait (HTMT) ratio of the correlations was used for the discriminant validity assessment proposed by Henseler et al. (2015). The threshold value of the HTMT ratio is lower than 0.85 or 0.90. Table 3 exhibits the HTMT values of all constructs, which are less than the threshold value 0.85 or 0.90 and validated the constructs.

**TABLE 3:** Discriminant validity (HTMT).

Sr. no	Constructs	CC	CLE	CM	EC	FA	TS
(1)	Curriculum content	0.716					
(2)	Campus learning environment (CLE)	0.530	0.711				
(3)	Curriculum material (CM)	0.559	0.676	0.765			
(4)	Entrepreneurial competencies (EC)	0.402	0.553	0.475	0.711		
(5)	Feedback and assessment (FA)	0.436	0.738	0.598	0.478	0.802	
(6)	Teaching strategies (TS)	0.529	0.716	0.759	0.459	0.659	0.805

HTMT, heterotrait-to-monotrait ratio.

We measured and solved the collinearity problems through structural equation modeling (SEM). The indicator used for collinearity testing is the variance inflation factor (VIF) (Huang, 2021). The threshold value for the VIF is less than 5. Table 4 indicates that the VIF range is between 1.542 and 3.066. It shows there was no collinearity problem between dimensions. Common method bias was found through Harman’s one-factor analysis (Mittal and Dhar, 2015). One principal component factor was applied for factor analysis of all constructs. It is recommended (Podsakoff, 2003) that results should not cross the threshold of 50% for unrotated factor analysis, while the result of Harman’s one-factor analysis showed 38.3%. It was found that there was no issue of CMB.

**TABLE 4:** Collinearity analysis and model fit.

Dimensions	VIF-CLE	VIF-EC	Model fit	
CC	1.542	1.597	Saturated model	
CM	2.554	2.651	SRMR	0.054
FA	1.840	2.384	NFI	0.878
TS	2.918	3.066	rms Theta	0.096
CLE		3.000		

CC, curriculum contents; curriculum material; TS, teaching strategies; FA, feedback and assessment; CLE, campus learning environment; EC, entrepreneurial competencies; SRMR, NFI.

In total, three main indicators, namely, SRMR, NFI, and RMS_theta, were used for testing the model fit in PLS-SEM. The threshold values for SRMR, NFI, and RMS_theta are less than 0.08, above 0.90, and less than 0.12, respectively (Bentler and Bonett, 1980; Henseler et al., 2014). Table 4 indicates that the SRMR, NFI, and RMS_theta indexes are 0.054, 0.878, and 0.096, respectively. All three major indicators for model fit indices showed that the model was reasonably well fitted in general. The collinearity and model fit analysis details are presented in Table 4.

The explanatory power of the model was assessed based on the *R*^2^ value. The range of *R*^2^ values is from 0 to 1. The explanatory power threshold values 0.75, 0.50, and 0.25 are considered robust, moderated, and feeble. Table 5 shows that the campus learning environment has a strong explanatory power, while entrepreneurial competencies have moderated explanatory power. Therefore, the model explains the latent variables very well and has a reasonable degree of explanatory power.

**TABLE 5:** *R*-square value.

Latent variables	*R* square	*R* square adjusted
Campus learning environment	0.667	0.667
Entrepreneurial competencies	0.335	0.335

## Descriptive statistics

The survey respondents were analyzed using descriptive statistics presented in Table 6. As mentioned earlier, the responses were taken on a seven-point Likert scale. The range of mean scores of responses is 4.156–5.260. The standard deviation range was recorded from 0.899 to 1.513.

**TABLE 6:** Descriptive statistics.

Constructs	*N*	Minimum	Maximum	Mean	Std. deviation
Curriculum content	461	1.00	7.00	5.024	1.129
Curriculum material	461	1.00	7.00	5.105	1.299
Teaching strategies	461	1.00	7.00	5.150	1.411
Feedback and assessment	461	1.00	7.00	4.156	0.783
Campus learning environment	461	1.00	7.00	5.260	1.513
Entrepreneurial competencies	461	1.00	7.00	5.211	1.417

N, number.

### Hypothesis testing

The study tested the hypothesis through a bootstrapping mechanism (5,000) by statistically efficient software SmartPLS (PLS-SEM 3.3.3) (Hair et al., 2016). Table 7 shows the direct and indirect effects of variables mentioned in the theoretical framework, along with *t*-values and *P*-values. The results exposed that the curriculum content positively and significantly influenced entrepreneurial competencies (β = 0.133, *p* < 0.05); thus, H1a was approved. However, the curriculum material did not positively and significantly influence entrepreneurial competencies (β = 0.011, *p* > 0.05); thus, H1b was not approved. Likewise, teaching strategies were also not positively and significantly influenced entrepreneurial competencies (β = −0.03, *p* > 0.05). Thus, H1c was not supported. Similarly, feedback and assessment did not have a positive influence on entrepreneurial competencies (β = 0.125, *p* > 0.05). Therefore, H1d was not approved.

**TABLE 7:** Direct relations.

Hypothesis	Direct relations	Coefficients	Mean	*SD*	*T* statistics	*P*-values	Results
H1a	CC → EC	0.133	0.134	0.052	2.54	0.011	Sig
H1b	CM → EC	0.11	0.109	0.073	1.495	0.135	Insig
H1c	TS → EC	−0.03	−0.031	0.076	0.388	0.698	Insig
H1d	FA → EC	0.125	0.125	0.07	1.767	0.077	Insig
H2a	CC → CLE	0.121	0.121	0.037	3.247	0.001	Sig
H2b	CM → CLE	0.171	0.17	0.054	3.19	0.001	Sig
H2c	TS → CLE	0.422	0.422	0.046	9.169	0.000	Sig
H2d	FA → CLE	0.319	0.322	0.071	4.471	0.000	Sig
H3	CLE → EC	0.244	0.247	0.057	4.322	0.000	Sig
Control variables	Gender → EC	0.117	0.117	0.041	2.864	0.004	
	Age → EC	0.115	0.114	0.037	3.086	0.002	

CC, curriculum contents; curriculum material; TS, teaching strategies; FA, feedback and assessment; CLE, campus learning environment; EC, entrepreneurial competencies; SD, standard deviation.

Moreover, the curriculum content had a positive and significant effect on the campus learning environment (β = 0.121, *p* < 0.05), and H2a was approved. Similarly, the curriculum material had a positive and significant effect on the campus learning environment (β = 0.171, *p* < 0.05), and H2b was supported. Likewise, teaching strategies had a positive and significant effect on the campus learning environment (β = 0.422, *p* < 0.05), and H2c was approved. Furthermore, feedback and assessment had a positive and significant effect on the campus learning environment (β = 0.319, *p* < 0.05), and H2d was accepted. Moreover, the campus learning environment had a positive and significant connection with entrepreneurial competencies (β = 0.244, *p* < 0.05), and H2b was supported. In addition, we also measured two control variables, namely, gender and age. Both gender and age had direct influence on entrepreneurial competencies (β = 0.117, *p* < 0.05; β = 0.115, *p* < 0.05).

### Mediation effects

**FIGURE 2:**
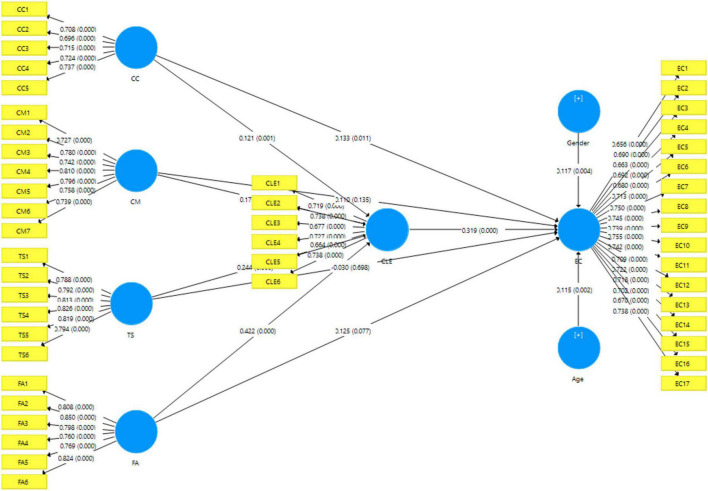
Structural relations between constructs.

To test campus learning environment-mediating effects, we checked the indirect effects of the curriculum content, curriculum material, teaching strategies, feedback and assessment, and entrepreneurial competencies; the results are exhibited in Table 8. An indirect effect was found a positive and significant influence of the curriculum content, curriculum material, teaching strategies, and feedback and assessment on entrepreneurial competencies (β = 0.39, *p* < 0.05, β = 0.55, *p* < 0.05, β = 0.78, *p* < 0.05, and β = 0.135, *p* < 0.05). Thus, the campus learning environment mediated the relationship between curriculum content, curriculum material, teaching strategies, and feedback and assessment, and entrepreneurial competencies. In order to find the partial mediation or full mediation of curriculum material was significant by direct and indirect effects. We find that curriculum material had partially mediated the effects on entrepreneurial competencies through campus learning environment while curriculum content, teaching strategies, and feedback and assessment were only indirectly influenced entrepreneurial competencies which have full mediation. As indicated in Table 8, hypotheses H4b-H4d were accepted. Figure 2 and Table 8 present details of the results.

**TABLE 8:** Indirect relations.

Hypothesis	Direct relations	Coefficients	Mean	*SD*	*T* statistics	*P*-values	Results
H4a	CC → CLE → EC	0.039	0.039	0.015	2.569	0.010	Sig
H4b	CM → CLE → EC	0.055	0.055	0.022	2.431	0.015	Sig
H4c	TS → CLE → EC	0.078	0.08	0.026	3.015	0.003	Sig
H4d	FA → CLE → EC	0.135	0.135	0.032	4.254	0.000	Sig

CC, curriculum contents; curriculum material; TS, teaching strategies; FA, feedback and assessment; CLE, campus learning environment; EC, entrepreneurial competencies; SD, standard deviation.

### Discussion

The majority of research on entrepreneurship education has been conducted in advanced countries (Seikkula-Leino, 2011; Kirkley, 2017; Iwu et al., 2019). In this research, we contributed some work for academicians and practitioners by producing knowledge in an emerging nation. First, this study investigated the influence of the entrepreneurial curriculum on entrepreneurial competencies. Second, we investigated the direct influence of the entrepreneurial curriculum on the campus learning environment. Third, the present study assessed the direct influence of the campus learning environment on entrepreneurial competencies. Fourth, this study explored the intervening role of the campus learning environment in the relationship between entrepreneurial curriculum and entrepreneurial competencies.

At first, the current study explored the direct connection between the curriculum content, curriculum material, teaching strategies, and assessment and feedback, and entrepreneurial competencies. The results revealed that the entrepreneurial curriculum content has a positive association with entrepreneurial competencies and supported our intuitions in hypothesis H1a. The results were consistent with previous study findings that the curriculum content influenced entrepreneurial competencies (Shirokova et al., 2017; Gieure et al., 2019). Lee et al. (2018) conducted a study on 927 university students to identify whether the university-based curriculum practices have an influence on entrepreneurial competencies. However, the results revealed that entrepreneurial curriculum material, teaching strategies, and assessment and feedback were not associated significantly and positively with the entrepreneurial competencies, and our hypotheses H1b, H1c, and H1d were disapproved. The results were constant with findings of previous studies (Igbokwe-Ibeto et al., 2018; Akhmetshin et al., 2019). Akhmetshin et al. (2019) explored the entrepreneurial curriculum was not aligned with entrepreneurial competencies in Russia and suggested revisions in the entrepreneurial curriculum. The possible reason could be that certain components of the entrepreneurial curriculum needed revisions in China.

Second, the current study investigated the entrepreneurial curriculum has a positive effect on the campus learning environment. The results found that the entrepreneurial curriculum (curriculum content, curriculum material, teaching strategies, and feedback and assessment) is positively and significantly associated with the campus learning environment, which endorses our hypotheses H2a–H2d. The results were also in line with previous studies results that showed the entrepreneurial curriculum is associated with the campus learning environment (Byun et al., 2018; Olutuase et al., 2020). A study conducted in America confirmed that the entrepreneurial curriculum and the campus learning environment are associated (Miller and Acs, 2017). Therefore, it was concluded that an effective entrepreneurial curriculum might engage students better in the campus learning environment.

Third, the study explored the direct effect of the campus learning environment on entrepreneurial competencies. The results of the present study revealed that the campus learning environment has a significant and positive effect on entrepreneurial competencies and approved our hypothesis H3. Previous studies also confirmed our study results that the campus learning environment is associated with entrepreneurial competencies (Beliaeva et al., 2017; Secundo et al., 2017). Hasan et al. (2017) conducted a study in Bangladesh among university students and found that university education was positively associated with entrepreneurial development. Therefore, we may conclude that the campus learning environment has a positive role to enhance entrepreneurial competencies among students.

Last, this study measured the mediating role of the campus learning environment between the entrepreneurial curriculum and entrepreneurial competencies. The results confirmed that the campus learning environment mediated the relationship between the entrepreneurial curriculum (curriculum content, curriculum material, teaching strategies, and feedback and assessment) and entrepreneurial competencies, which approved our hypotheses H4a–H4d. These findings are in line with past research conducted on exploring the mediating effect of the entrepreneurial education on entrepreneurial attitudes (Barnes and de Villiers Scheepers, 2018; Lu et al., 2021; Luo et al., 2022; Valencia-Arias et al., 2022). Furthermore, Choi and Markham (2019) discussed the significance of the campus learning environment role to develop entrepreneurial competencies among the students as per the need of industry. Winkler et al. (2018) also explored using action research as a catalyst to constantly improve the entrepreneurship education environment based on a deeper understanding of students’ needs to build entrepreneurial competencies. We may therefore conclude that this finding is the novelty of our research and the latest contribution in field of entrepreneurial education and competencies.

### Conclusion

This study aimed to measure the influence of the entrepreneurial curriculum (curriculum content, curriculum material, teaching strategies, and feedback and assessment) on entrepreneurial competencies. Furthermore, this study investigated the influence of the entrepreneurial curriculum (curriculum content, curriculum material, teaching strategies, and feedback and assessment) on the campus learning environment. Similarly, the present study assessed the influence of the campus learning environment on entrepreneurial competencies. Finally, this study also explored the intervening role of the campus learning environment in the relationship between entrepreneurial curriculum (curriculum content, curriculum material, teaching strategies, and feedback and assessment) and entrepreneurial competencies.

By and large, this study exposed a statistically significant and positive correlation between the entrepreneurial curriculum content and entrepreneurial competencies among the pre-service vocational teachers. In addition, this study found no significant relationship between the entrepreneurial curriculum material, teaching strategies, and entrepreneurial competencies. The study also explored the significant and positive correlation between the entrepreneurial curriculum content, curriculum material, teaching strategies, feedback and assessment, and campus learning environment. Furthermore, it was also found that the campus learning environment and entrepreneurial competencies are positively and significantly correlated. For an indirect relationship, we find that the campus learning environment mediated the relationship between entrepreneurial curriculum content, curriculum material, teaching strategies, feedback and assessment, and entrepreneurial competencies of pre-service vocational teachers.

The finding of this research could be interpreted as follows: (1) It is concluded that the entrepreneurial curriculum content is a predictor of entrepreneurial competencies. Moreover, the educational program with the entrepreneurial curriculum material, teaching strategies, and feedback and assessment required some urgent changes for their influential role in developing entrepreneurial competencies. (2) The entrepreneurial curriculum content, curriculum material, teaching strategies, and feedback and assessment were associated with the campus learning environment. It was also concluded that effective entrepreneurial curriculum delivery might give a better output when it works with the campus learning environment. (3) The campus learning environment correlated with entrepreneurial competencies, and we may conclude that the campus learning environment plays a vital role in shaping entrepreneurial competencies. 4) It was concluded that campus learning environment plays a vital along with the entrepreneurial curriculum content, curriculum material, teaching strategies, and feedback and assessment to enhance entrepreneurial competencies. It can be concluded that the entrepreneurial curriculum works through the campus learning environment in shaping entrepreneurial competencies among students.

### Implications

The initial step is key to understanding critical entrepreneurial competencies and knowing how the flow theory can energize the basic insights to generate new ventures. This study revealed certain theoretical and practical implications, such as entrepreneurial competencies, could be developed by combining the entrepreneurial curriculum and conducive campus learning environment. The results confirmed the theory of plane behavior and human capital theory that students must have been given the vision to create new ventures. This study has practical implications, which may help enhance entrepreneurial competencies. This research may help university management understand the weaker curriculum elements and get guidelines to change them. The university should bring about changes in the curriculum material, teaching strategies, and feedback and assessment. University management ensures the provision of the resources to maintain the campus learning environment, which is helpful to develop entrepreneurial competencies among pre-service vocational teachers. Moreover, through the results, university teachers may understand the significance of the curriculum components, such as curriculum content, curriculum material, teaching strategies, and feedback and assessment approaches toward building a conducive campus learning environment, which supports to build entrepreneurial competencies among pre-service vocational teachers. Similarly, from the outcomes of the study, curriculum developers can comprehend the effective entrepreneurial components curriculum content, curriculum material, teaching strategies, and feedback and assessment approaches and bring effective changes in these components to enhance the entrepreneurial competencies among pre-service vocational teachers.

### Limitations and future research direction

The study has few limitations that may influence the interpretation of results. The participants in this study are from a single emerging country (China), which may contain a cultural bias and limit generalization of the findings to a broader group of people. Additional studies are required in different cultural contexts to ensure the validity of the results. Second, we collected the data from students of the vocational education teaching program only, which might cause bias in generalizing results to students from other disciplines. This study has only considered the campus learning environment as a mediating variable, while other mediating constructs, such as information technology skills, digital competencies, and student engagement, could also be practically explored. It would be interesting if future research used information technology skills and student engagement as mediator variables in the relationship between the entrepreneurial curriculum and campus learning environment.

## Data availability statement

The original contributions presented in this study are included in the article/Supplementary material, further inquiries can be directed to the corresponding author.

## Ethics statement

The studies involving human participants were reviewed and approved by Guangzhou University, China. The patients/participants provided their written informed consent to participate in this study.

## Author contributions

All authors listed have made a substantial, direct, and intellectual contribution to the work, and approved it for publication.
