# Supplementary material for: Impact of entrepreneurial curriculum on entrepreneurial competencies among students: The mediating role of the campus learning environment in higher education
Source: Front Psychol. 2022 Sep 15;13:950440. doi: 10.3389/fpsyg.2022.950440 (PMC9522719; doi:10.3389/fpsyg.2022.950440)
Supplement: Supplementary file 1 [file Data_Sheet_1.docx]

**Appendix A**

**Questionnaire**

| **Entrepreneurial Curriculum** | | | | | | | | | | |  |
| --- | --- | --- | --- | --- | --- | --- | --- | --- | --- | --- | --- |
| **Sr. No** | **Statements** | | **Strongly Disagree** | **Disagree** | **Little Disagree** | **Neutral** | **Little Agree** | **Agree** | **Strongly Agree** | |  |
| **Curriculum Content** | | | | | | | | | | |  |
| 1 | I have acquired sufficient content knowledge about entrepreneurship. | | 1 | 2 | 3 | 4 | 5 | 6 | 7 | |  |
| 2 | I can use (entrepreneurship content knowledge) as the path of thinking. | | 1 | 2 | 3 | 4 | 5 | 6 | 7 | |  |
| 3 | I have sufficient knowledge of planning entrepreneurial projects. | | 1 | 2 | 3 | 4 | 5 | 6 | 7 | |  |
| 4 | The teacher provides detailed information about entrepreneurship competencies. | | 1 | 2 | 3 | 4 | 5 | 6 | 7 | |  |
| 5 | The teachers made it clear right from the start that they expect us to become entrepreneurs. | | 1 | 2 | 3 | 4 | 5 | 6 | 7 | |  |
| **Curriculum Material** | | | | | | | | | | |  |
| Our teacher | | | | | | | | | | |  |
| 1 | uses audio, video and well devised material about entrepreneurship in the class. | | 1 | 2 | 3 | 4 | 5 | 6 | 7 | |  |
| 2 | uses material of entrepreneurial education that enhances my motivation to develop entrepreneurial competencies. | | 1 | 2 | 3 | 4 | 5 | 6 | 7 | |  |
| 3 | allows to practice entrepreneurial activities inside the classroom. | | 1 | 2 | 3 | 4 | 5 | 6 | 7 | |  |
| 4 | uses innovative material that help me understand the concept of entrepreneurship better. | | 1 | 2 | 3 | 4 | 5 | 6 | 7 | |  |
| 5 | used material in the class which was meaningful for me in using information technology in the context of entrepreneurial competencies. | | 1 | 2 | 3 | 4 | 5 | 6 | 7 | |  |
| 6 | used material in the class was meaningful for me in using information technology in the context of entrepreneurial education. | | 1 | 2 | 3 | 4 | 5 | 6 | 7 | |  |
| 7 | The material I used in the class induced interest and motivation in me which is helpful to develop entrepreneurial competencies. | | 1 | 2 | 3 | 4 | 5 | 6 | 7 | |  |
| **Teaching Strategies** | | | | | | | | | | |  |
| Our teachers | | | | | | | | | | |  |
| 1 | keep me motivated and engaged in entrepreneurial learning. | | 1 | 2 | 3 | 4 | 5 | 6 | 7 | |  |
| 2 | provide with a conducive entrepreneurial learning environment. | | 1 | 2 | 3 | 4 | 5 | 6 | 7 | |  |
| 3 | facilitate me in entrepreneurial learning. | | 1 | 2 | 3 | 4 | 5 | 6 | 7 | |  |
| 4 | use various teaching strategies during teaching. | | 1 | 2 | 3 | 4 | 5 | 6 | 7 | |  |
| 5 | meaningful interaction between students and teachers beyond the classroom are ensured. | | 1 | 2 | 3 | 4 | 5 | 6 | 7 | |  |
| 6 | used new and updated teaching strategies related to entrepreneurial competencies. | |  |  |  |  |  |  |  | |  |
| **Feedback and Assessment** | | | | | | | | | | |  |
| 1 | Our teachers give constructive feedback on my progress. | | 1 | 2 | 3 | 4 | 5 | 6 | 7 | |  |
| 2 | The teachers make a real effort to understand and solve challenges that I might be facing with my work. | | 1 | 2 | 3 | 4 | 5 | 6 | 7 | |  |
| 3 | The teachers invest a lot of time into commenting on my work. | | 1 | 2 | 3 | 4 | 5 | 6 | 7 | |  |
| 4 | Feedback from the teachers was helpful and timely. | | 1 | 2 | 3 | 4 | 5 | 6 | 7 | |  |
| 5 | I am assessed on how well I can apply what I have learned to new situations. | | 1 | 2 | 3 | 4 | 5 | 6 | 7 | |  |
| 6 | My teachers ask questions on how well I can integrate knowledge and skills acquired in a course. | | 1 | 2 | 3 | 4 | 5 | 6 | 7 | |  |
| **Campus Learning Environment** | | | | | | | | | | |  |
| 1 | I am able to discuss topics of broader intellectual interest with teachers about entrepreneurship. | | 1 | 2 | 3 | 4 | 5 | 6 | 7 | |  |
| 2 | I am able to participate with the entrepreneurial activities organized the campus. | | 1 | 2 | 3 | 4 | 5 | 6 | 7 | |  |
| 3 | I have acquired an in-depth knowledge about entrepreneurship from campus environment. | | 1 | 2 | 3 | 4 | 5 | 6 | 7 | |  |
| 4 | I strive for excellence in my academic / professional studies in campus life. | | 1 | 2 | 3 | 4 | 5 | 6 | 7 | |  |
| 5 | University environment is conducive for learning. | | 1 | 2 | 3 | 4 | 5 | 6 | 7 | |  |
| 6 | My teachers provide opportunities for interaction in class | | 1 | 2 | 3 | 4 | 5 | 6 | 7 | |  |
| 7 | Campus environment create opportunities for learning entrepreneurial competencies. | |  |  |  |  |  |  |  | |  |
| **Entrepreneurial Competencies** | | | | | | | | | | |  |
| After completion of my education program, I would; | | | | | | | | | | |  |
|  | Make rational decision in the organization | 1 | | 2 | 3 | 4 | 5 | 6 | | 7 | |
|  | Develop long-term trustable relationships with colleagues. | 1 | | 2 | 3 | 4 | 5 | 6 | | 7 | |
|  | Negotiate with others. | 1 | | 2 | 3 | 4 | 5 | 6 | | 7 | |
|  | Maintain a personal network of professional contacts. | 1 | | 2 | 3 | 4 | 5 | 6 | | 7 | |
|  | Integrate ideas, issues and observations into more general contexts. | 1 | | 2 | 3 | 4 | 5 | 6 | | 7 | |
|  | Monitor progress toward objectives in risky actions. | 1 | | 2 | 3 | 4 | 5 | 6 | | 7 | |
|  | Explore new ideas. | 1 | | 2 | 3 | 4 | 5 | 6 | | 7 | |
|  | Plan the operations of the institution. | 1 | | 2 | 3 | 4 | 5 | 6 | | 7 | |
|  | Organize resources. | 1 | | 2 | 3 | 4 | 5 | 6 | | 7 | |
|  | Lead subordinates. | 1 | | 2 | 3 | 4 | 5 | 6 | | 7 | |
|  | Motivate people. | 1 | | 2 | 3 | 4 | 5 | 6 | | 7 | |
|  | Determine long-term issues, problems, or opportunities. | 1 | | 2 | 3 | 4 | 5 | 6 | | 7 | |
|  | Prioritize work in alignment with organizational goals. | 1 | | 2 | 3 | 4 | 5 | 6 | | 7 | |
|  | Induce constructive changes in the institution to comply with the future challenges | 1 | | 2 | 3 | 4 | 5 | 6 | | 7 | |
|  | Evaluate results against strategic goals. | 1 | | 2 | 3 | 4 | 5 | 6 | | 7 | |
|  | Determine strategic actions by weighing costs and benefits. | 1 | | 2 | 3 | 4 | 5 | 6 | | 7 | |
|  | Commit to long-term business goals of my own institution. | 1 | | 2 | 3 | 4 | 5 | 6 | | 7 | |
